# B-cell receptor physical properties affect relative IgG1 and IgE responses in mouse egg allergy

**DOI:** 10.1038/s41385-022-00567-y

**Published:** 2022-09-16

**Authors:** Christopher C. Udoye, Christina N. Rau, Sarah M. Freye, Larissa N. Almeida, Sarah Vera-Cruz, Kai Othmer, Rabia Ü. Korkmaz, Ann-Katrin Clauder, Timo Lindemann, Markus Niebuhr, Fabian Ott, Kathrin Kalies, Andreas Recke, Hauke Busch, Anke Fähnrich, Fred D. Finkelman, Rudolf A. Manz

**Affiliations:** 1 Institute for Systemic Inflammation Research, University of Lübeck, Lübeck, Germany; 2 Institute for Anatomy, University of Lübeck, Lübeck, Germany; 3 Medical Systems Biology Division, Lübeck Institute of Experimental Dermatology and Institute for Cardiogenetics, University of Lübeck, Ratzeburger Allee 160, 23562 Lübeck, Germany; 4 Department of Dermatology, Allergology and Venereology, University off Lübeck, Lübeck, Germany; 5 Division of Allergy, Immunology and Rheumatology, Department of Internal Medicine, University of Cincinnati College of Medicine and the Division of Immunobiology, Cincinnati Children’s Hospital Medical Center, Cincinnati, OH USA

## Abstract

Mutated and unmutated IgE and IgG play different and partly opposing roles in allergy development, but the mechanisms controlling their relative production are incompletely understood. Here, we analyzed the IgE-response in murine food allergy. Deep sequencing of the complementary-determining region (CDR) repertoires indicated that an ongoing unmutated extrafollicular IgE response coexists with a germinal center response, even after long-lasting allergen challenges. Despite overall IgG1-dominance, a significant proportion of clonotypes contained several-fold more IgE than IgG1. Clonotypes with differential bias to either IgE or IgG1 showed distinct hypermutation and clonal expansion. Hypermutation rates were associated with different physiochemical binding properties of individual B-cell receptors (BCR). Increasing BCR signaling strength inhibited class switching from IgG1 to IgE in vitro, preferentially constraining IgE formation. These data indicate that antigen-binding properties of individual BCRs determine differential IgE hypermutation and IgE versus IgG1 production on the level of single B-cell clones.

## Introduction

Human and murine B cells can produce a variety of different immunoglobulin (Ig) classes (IgM, IgD, IgG, IgE, and IgA) using different constant regions of the heavy chain (CH) regions^1,2^. Membrane-bound Igs serve as the BCR and determine the antigen-specificity of the B-cell response^3^, with different Ig subclasses having distinct but overlapping signaling properties^4–8^. In their soluble form secreted by plasma cells, the different Ig classes have different effector functions and thus play distinct roles in immune protection and disease^2,9^. Although IgE has the shortest half-life and shows the lowest serum concentration of all Ig subclasses, it plays important roles for immune protection against helminths and has a key role in type-1 atopic disorders^10–12^. IgE production and serum levels seem to be limited by multiple mechanisms, including limited formation and/or survival of IgE+ B cells^6,13^. IgE expression requires an irreversible DNA rearrangement, called class switch recombination, that can occur in activated mature B cells^14^. Naive IgD+/IgM+ B cells can switch to IgE either directly, or sequentially through an intermediate IgG+ B-cell stage. Both processes involve directed deletional loop-out-mediated recombination between two highly repetitive switch regions, which generates short-lived switch circles that differ for direct and sequential switching^15,16^. Class switch does not affect the variable chains that contain the CDR responsible for antigen recognition^16^. Class switch can occur in both extrafollicular locations and in germinal centers. However, the extrafollicular response yields B-cell clones with comparably low hypermutation rates^17,18^.

Within germinal centers, activated B-cell clones proliferate and hypermutate their variable region genes, including the CDRs. Following selection of B-cell clones expressing antibodies of higher affinities, memory B cells, and plasma cells are released from germinal centers; these can persist for years to decades^19–21^. High-affinity clones are directly attracted to the plasma cell fate, while clones starting with lower affinities enter the germinal center reaction to become memory B cells^22^.

Next-generation sequencing (NGS) of the variable region of the BCR repertoires has brought deep insights into these processes and their contribution to the pathogenesis of allergic and autoimmune diseases^23–26^. In different murine models and patients, persistent IgE production has been attributed either to the formation of long-lived IgE+ plasma cells^27,28^ or reactivation and class switching of IgG1+ memory B cells^29–31^. In humans, long-lived plasma cells seem to play a more prominent role for persistent IgE production than has been reported for most mouse models^32^. The generation of long-lived IgE+ plasma cells seems to require repeated antigen/allergen challenges^27^.

Not all IgE mediates allergic symptoms. Highly mutated, high-affinity IgE can activate mast cells and cause allergic anaphylaxis. In contrast, less mutated, low-affinity IgE does not activate mast cells and can prevent anaphylaxis by competing with high-affinity IgE for binding to Fcε receptors^33–35^.

Although IgE, murine IgG1, and human IgG4 are all produced through T-cell-dependent type 2 immune responses involving interleukin (IL)-4 or IL-13^36^, IgE and IgG1 (IgG4) have opposing roles in allergy development^37^. Allergen-induced crosslinking of IgE bound to the FcεRI on mast cells can induce type-1 hypersensitivity reactions. In contrast, allergen-specific IgGs can neutralize absorbed allergen, thereby inhibiting IgE-mediated diarrhea and anaphylaxis^38^. Therefore, the ratio between IgG and IgE production as well as the generation of poorly mutated versus hypermuted IgE can be relevant for allergy development, in humans and in mice.

Here, we studied the formation of unmutated and hypermutated IgE and IgG1, their repertoires, and their clonal relationships in a mouse model of food allergy to hen´s egg, using repeated allergen challenge over a relatively long period of time.

## Results

### Diarrhea and allergen-induced temperature drop are associated with low allergen-specific IgG1 to IgE ratios

**Fig. 1:**
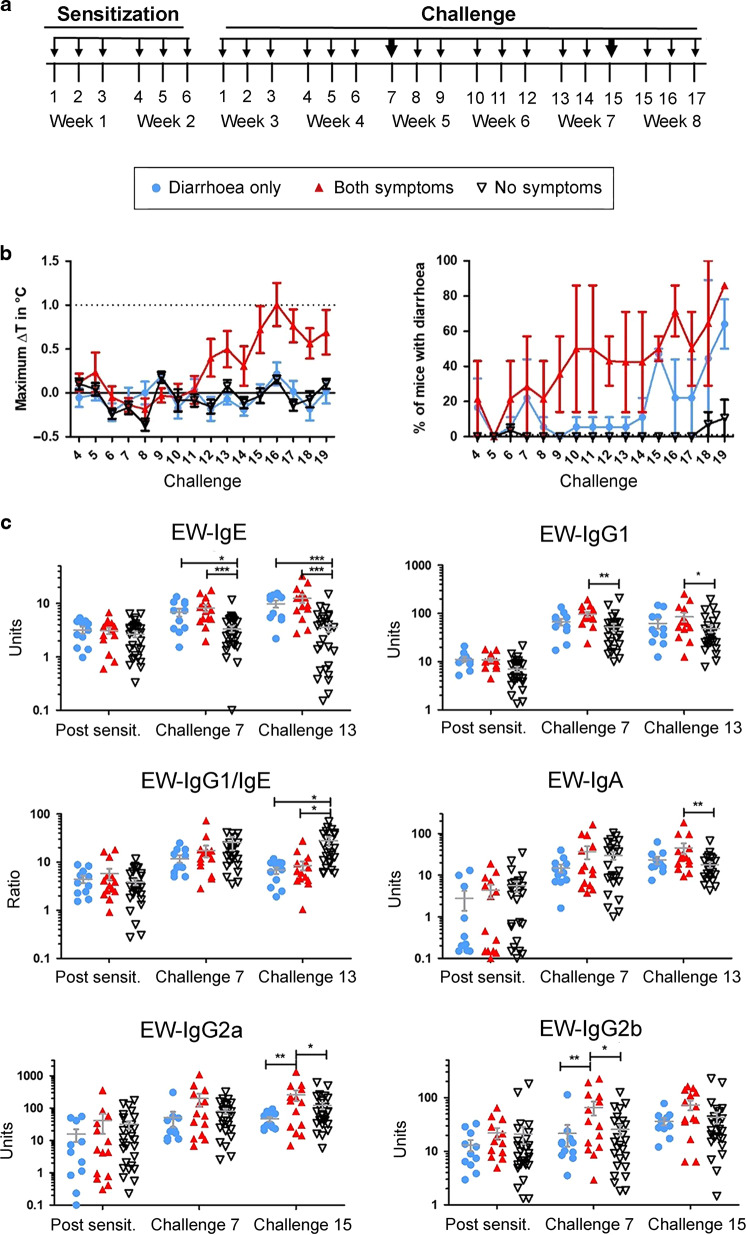
The food allergy model. Mice were sensitized to and challenged with EG/EYP and analyzed for the development of allergic symptoms. **a** Experimental outline. **b** Based on the development of symptoms, mice were categorized into different groups: mice that had at least 2× diarrhea but never a temperature drop ≥ 1 °C in response to a challenge (blue dot, diarrhea only; *n* = 11); mice that had at least 1× diarrhea and 2× a maximum temperature drop (max. Δ*T*) ≥ 1 °C in response to a challenge (red triangle, both symptoms; *n* = 14) and mice that never showed diarrhea or a max. Δ*T* ≥ 0.5 °C (black triangle No symptoms; *n* = 28), as indicated. The average drop in body temperature (left) and the occurrence of diarrhea (right) are shown. Data presented as mean ± SEM. **c** Mice were bled and EW-specific antibodies were measured by ELISA. EW-specific Ig subclasses and the ratio of IgG1 to IgE are shown. Pooled serum from EW/EYP-allergic mice was used as a standard that was defined as having 100 units of each Ab. Each symbol represents data form one mouse. Total number of mice: 53. No sample was excluded from the analysis. Statistics: two-way ANOVA with Bonferroni post test; not significant (no mark): *p* > 0.05 *p < 0.05, **p < 0.01, ***p < 0.001. Mean ± SEM is shown.

To investigate the development of allergen-specific antibodies and murine food allergy, mice were sensitized to hen’s egg by intra-tracheal treatment with egg white (EW)/egg yolk plasma (EYP) followed by repeated oral gavage (o.g.) with EW and EYP^39^. Starting at week four, mice were examined for diarrhea and hypothermia in response to o.g. egg inoculation (Fig. 1a). Depending on the individual response, mice could be assigned into three different groups characterized by the development of either diarrhea alone, diarrhea, and hypothermia, or neither (Fig. 1b). The development of diarrhea and hypothermia was strongly associated with increased EW-specific IgE and IgG1 titers (Fig. 1c). IgA and IgG2a anti-EW titers were also highest for mice that had both diarrhea and hypothermia at challenge 13 and 15, respectively, but not after challenge 7, while IgG2b anti-EW titers were highest for mice that had both diarrhea and hypothermia after challenge 7, but not challenge 15. Consistent with a protective effect of IgG1^38^, allergen-specific IgG1 to IgE ratios averaged more than tenfold higher in mice with neither disease feature as compared to mice that had one or both features after 13 challenges.

### Follicular and extrafollicular responses contribute to IgE production

In order to investigate the IgE and IgG1 repertoires, samples were taken from the intestine-draining mesenteric lymph nodes (mLN) and bone marrow (BM) of two naive, and five allergic mice that had developed a temperature drop ≥ 1 °C and diarrhea after seven weeks of repeated allergen challenges (13 o.g. egg inoculations). cDNA was generated from the individual samples, barcoded, and amplified, after which the BCR-repertoires encoding the antigen-binding VDJ-IgH-regions that were 5′ to Cε or Cγ1 sequences were analyzed by NGS (Fig. S1).

**Fig. 2:**
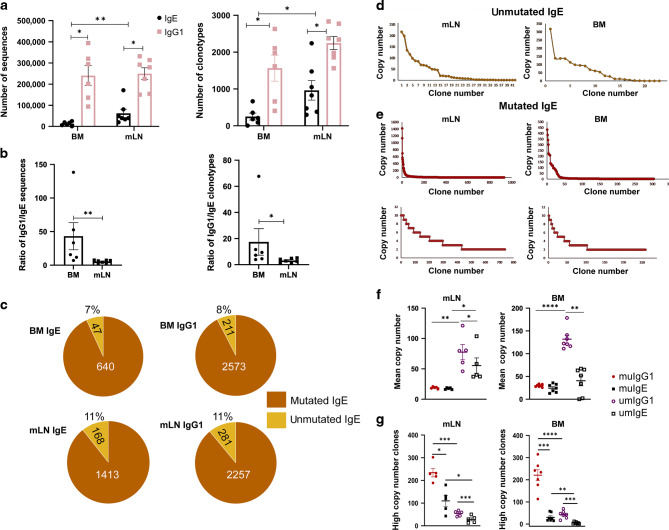
IgE and IgG1 sequences and clonotypes. cDNA from naive mice and allergic mice challenged o.g. 13 times with EG/EYP was analyzed by NGS. Individual clonotypes within the IgG1 and IgE IgH-repertoires were defined by unique VDJ-rearrangements. **a** Number of sequences and clonotypes in BM and mLN, as indicated. Colored bars represent the mean. **b** Ratios of IgG1 to IgE sequences and ratios of IgG1 to IgE clonotypes, as indicated. **c** Number and percentages of hypermutated and unmutated IgE and IgG1 clonotypes. Representative data from two samples from BM and mLN each are shown. **d** Clone size distribution of unmutated IgE clonotypes. Representative data from mLN and BM of one mouse are shown, as indicated. **e** Upper panel: clone size distribution of all mutated IgE clonotypes. Lower panel: Clone size distribution of mutated IgE clonotypes with a clone size of 10 or less. **f** Mean copy numbers. Each symbol represents the average copy numbers of mutated IgG1 clonotypes (muIgG1), mutated IgE clonotypes (muIgE), unmutated IgG1 clonotypes (umIgG1) and unmutated IgE clonotypes (umIgE) from the mLN or BM of one mouse, as indicated. **g** Number of high copy number clones, defined as clones with copy numbers greater than the average umIgE or umIgG1 copy number, respectively. a/b: samples from 5 allergic mice and 2 naive mice from one experiment were analyzed. One BM sample was excluded due to insufficient cDNA quality (mLN: *n* = 7, BM: *n* = 6). **c**–**f** samples from mLN of 5 allergic mice from one experiment and pooled samples from BM of 7 allergic mice from two experiments are shown (mLN: *n* = 5, BM: *n* = 7). No sample was excluded from the analysis. Total number of mice: 7. Statistics a-c: Mann Whitney’s nonparametric test for comparisons within the BM or mLN; Wilcoxon nonparametric matched paired test for comparisons between BM and mLN. Data presented as mean ± SEM. Statistics **d**–**g**: pairwise *t*-test. *p ≤ 0.05; **p ≤ 0.01; ***p ≤ 0.001; ****p ≤ 0.0001.

Between one and four hundred thousand sequences were analyzed for each sample (Table S1). IgG1 sequences were approximately four- and nineteen-times more abundant than IgE sequences (Fig. 2a). Individual BCR clonotypes were defined as sharing a unique VDJ rearrangement with conserved CDR3-IMGT anchors (cysteine C 104, tryptophan W 118 or phenylalanine F 118). IgE sequences and IgE clonotypes were approximately 30% less frequent in BM than in mLN. The ratios of IgG1/IgE sequences and of IgG1/IgE clonotypes were 5-8-fold higher in BM compared to mLN (Fig. 2b), indicating that the accumulation of IgE^+^ plasma cells within the BM is more tightly restricted than the accumulation of IgG1^+^ plasma cells in this tissue. This conclusion is consistent with results suggesting that IgE and IgG BCRs differently impact B-cell fate and the development of long-lived plasma cells^40,41^ and that IgG^+^ plasma cells have a better BM homing ability than IgE^+^ plasma cells^42^. While the majority of IgE and IgG1 clonotypes showed VDJ-hypermutation, VDJ-hypermutation was absent in approximately 7–12 % of IgE and IgG1 in mLN and BM clonotypes (Fig. 2c). Approximately half of these unmutated clonotypes were clonally expanded, with more than 50 copies per clones (Fig. 2d).

Mutated IgE clones were more abundant, but their clone size was much more variable. While a few mutated IgE clones had a huge clone size with copy numbers from 100 to >1000, the great majority of mutated IgE clones was present in copy numbers below 10 (Fig. 2e). Comparable numbers of unmutated and mutated IgE clonotypes were present for clonotypes present in copy numbers >50.

As a result of the differences in clone size distribution, unmutated IgE and IgG1 clonotypes had higher mean copy numbers than mutated clones (Fig. 2f), but clonotypes with high copy numbers (copy numbers ≥ the mean for unmutated IgE and IgG1) were more abundant among the mutated clonotypes (Fig. 2g).

These results are in accord with the co-existence of a follicular response and an ongoing recruitment of naive B cells into an extrafollicular response, with the former yielding hypermutated and highly selected IgE clonotypes and the latter forming unmutated IgE clonotypes that underwent relatively little clonal selection. This finding is consistent with a recent study indicating that an extrafollicular response contributes to chronic IgE production in patients suffering from allergic rhinitis^43^.

### BCR clonotypes vary in IgE to IgG1 ratios

The great majority of individual mutated and unmutated IgE sequences were also present as IgG1 sequences (Table 1). This is consistent with a close relationship between the IgE and IgG1 repertoires and the development of most secondary IgE responses from the IgG1 memory compartment^24,29,44^.

**Table 1:** IgE clonotypes.

Sample ID	Mutated		Umutated		Percentage mutated	
	Shared clones (no.)	Unique IgE clones (no.)	Shared clones (no.)	Unique IgE clones (no.)	Mutated shared (%)	Mutated unique (%)
mLN768	1139	465	129	22	89.8	95.4
mLN758	305	59	27	1	91.8	98.3
mLN769	562	97	46	2	92.4	97.9
mLN753	774	276	115	14	87.0	95.1
mLN777	710	121	78	13	90.1	90.2
BM82400	52	20	8	1	86.6	95.2
BM82416	11	5	1	0	91.6	100
BM777	129	22	9	1	93.4	95.6
BM758	160	20	17	1	90.3	95.2
BM82439	12	3	0	0	100	100
BM753	490	78	29	10	94.4	88.6
BM768	231	65	12	1	95.06	98.4

Clones are defined by identical VDJ-rearrangements. Clonotyes present in the IgE and IgG1 repertoires are defined as “shared clones”. Clonotyes present in the IgE repertoire but absent from the IgG repertoire are defined as “unique IgE clones”. The sample IDs from fife mLN samples and seven BM samples of individual mice are indicated.

**Fig. 3:**
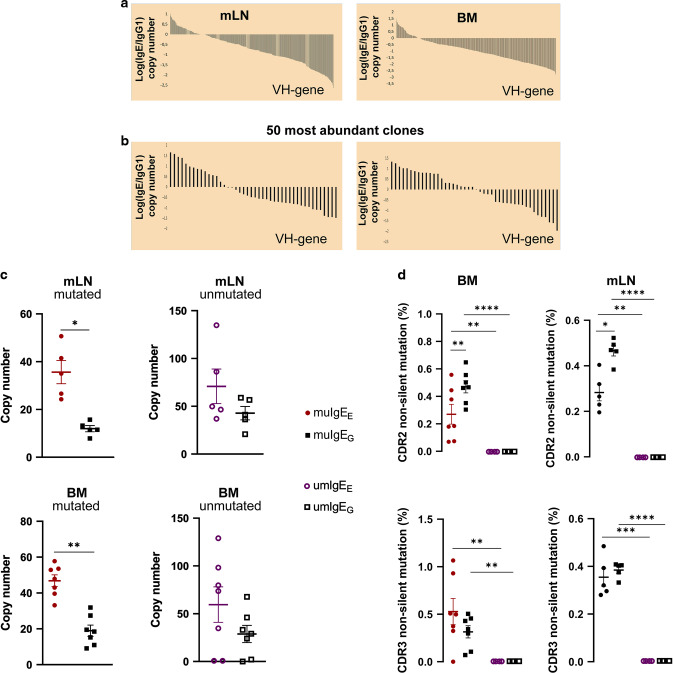
IgE_G_ and IgE_E_ clonotypes. Sequences from allergic mice were analyzed after 13 o.g. allergen challenges. **a** Shared clonotypes (VH-genes) were ordered in accordance to the ratios of their IgE to IgG1 copy numbers. Representative data from one mLN and one BM sample are shown. **b** Shared clonotypes from the 50 most abundant clonotypes. **c** Shared clones were classified as IgE-biased (IgE_E_, IgE/IgG copy number ratio of ≥ 2), or IgG1-biased (IgE_G_, IgG/IgE copy number ratio of ≥ 2). Copy numbers of mutated IgE_E_ (mIgE_E_), mutated IgE_G_ (mIgE_G_), unmutated IgE_E_ (umIgE_E_), and unmutated IgE_G_ (umIgE_G_) from BM and mLN are shown, as indicated. Each symbol represents the mean copy numbers form one mouse for the IgE subtype indicated. mLN samples from 5 allergic mice from one experiment and BM samples from 7 mice pooled from two independent experiments were analyzed (mLN: *n* = 5, BM: *n* = 7). No sample was excluded from the analysis. **d** Hypermutation rates of IgE_G_ and IgE_E_. Mean percentage of non-silent mutations within the IgH-CDR3 regions of mutated IgE_E_ (muIgE_E_), mutated IgE_G_ (muIgE_G_), unmutated IgE_E_ (umIgE_E_) and unmutated IgE_G_ (umIgE_G_) are shown for BM and mLN, as indicated. Data presented as mean ± SEM. Statistics: pairwise *t*-test. *p ≤ 0.05; **p ≤ 0.01; ***p ≤ 0.001; ****p ≤ 0.0001.

As expected from the overall excess of IgG1 compared to IgE sequences, most shared clonotypes showed a several-fold excess of IgG1 compared to IgE copies. However, other clones shared between IgE and IgG1 showed a strong bias towards IgE, containing 5 to 10-fold more IgE than IgG1 copies (Fig. 3a, b). Based on these data, clones found within the IgE repertoire were classified as IgE-biased (IgE_E_, with an IgE/IgG1 copy number ratio ≥ 2) or IgG1-biased (IgE_G_, with an IgG1/IgE copy number ratio ≥ 2).

As indicated by their copy numbers (clone size), IgE_E_ and IgE_G_ showed different levels of clonal expansion (Fig. 3c). Mutated IgE_E_ clone sizes averaged > 2-fold larger than mutated IgE_G_ clones in both mLN and BM. Clone sizes of unmutated IgE subtypes did not show significant differences. Consistent with the idea that the increased clonal expansion of mutated IgE_E_ compared to IgE_G_ is the consequence of differential selection within the germinal center, the two mutated IgE subtypes showed different hypermutation rates in the CDR regions (Fig. 3d).

In order to investigate whether IgE_E_ and IgE_G_ clones represent a unique feature of our allergy model, the BCR repertoires of the IgE and IgG1 compartments in BM of two naive mice were analyzed. The number of distinct clonotypes was low, as expected for unimmunized mice (Fig. S2). The few individual clonotypes and the high degree of variation is consistent with the undefined immune status and overall low-level immune stimulation of naive mice. The detectable IgE clonotypes showed also a bias for either IgE or IgG1. Next, we re-analyzed published sequences from mice infected with *Nippostrongylus brasiliensis*^31^, a helminth parasite that induces a strong IgE response. IgE_E_ and IgE_G_ were also present in the *N. brasiliensis*-infected mice, and within the infection model there were also mutated and unmutated IgE subtypes found (Fig. S2b, Table S2).

In our murine egg-allergy model, approximately 10–20% of IgE clonotypes were unique, i.e. not detectable as IgG1 clonotypes. But these unique clonotypes were present in comparably low copy numbers (Fig. S3).

Together, these data indicate that IgE_E_ and IgE_G_ clonotypes represent a common phenomenon, seen in naive, allergic and helminths infected mice. They show that the relative IgE to IgG1 production is controlled at the level of individual B-cell clones, with mutated IgE_E_ and IgE_G_ subjected to differential hypermutation and clonal selection. Hence, individual BCRs themselves seem to have a strong impact on the nature of the IgE response and its relationship to the IgG1 response.

### BCR properties affect the formation of extrafollicular and follicular IgE_E_ and IgE_G_

**Fig. 4:**
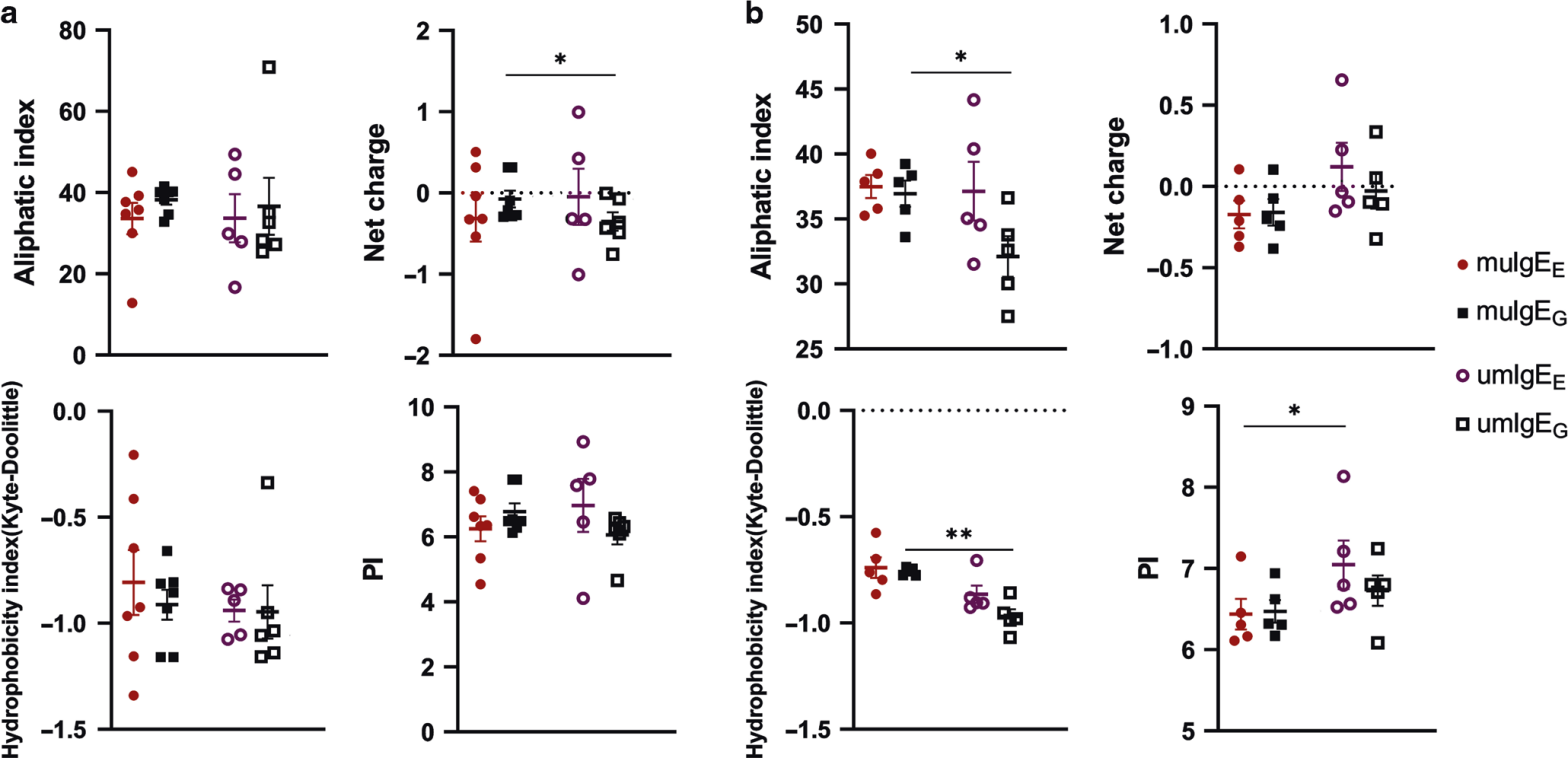
Physiochemical properties of IgE_G_ and IgE_E_. Sequences from mLN and BM of allergic mice were analyzed after 13 o.g. allergen challenges. Physiochemical properties of IgE_E_ and IgE_G_ clones were calculated using the “Peptide” package in the R program. Aliphatic index, hydrophobicity index according to Kyte-Doolittle, net charges and isoelectric point (PI), are shown for mutated IgE_E_ (muIgE_E_), mutated IgE_G_ (mIgE_G_), unmutated IgE_E_ (umIgE_E_) and unmutated IgE_G_ (umIgE_G_). mLN samples from 5 allergic mice from one experiment and BM samples from 7 mice pooled from two independent experiments were analyzed. Two BM samples did not contain detectable umIgE_E/_ umIgE_G_. **a** Data from BM (*n* = 5–7). **b** Data from mLN (*n* = 5). Each symbol represents the respective average ratios form one mouse. Data presented as mean ± SEM. Statistics: pairwise *t*-test. *p ≤ 0.05; **p ≤ 0.01.

Net charge, aliphatic index, hydrophobicity, and isoelectric points are important parameters that determine the antigen-binding priorities and polyreactivity of individual BCRs^45^. These physiochemical properties were calculated using the “Peptide” package in the R program. Overall, mutated IgE_E_ and IgE_G_ clonotypes exhibited similar physiochemical properties, indicating that they are not relevant for the generation of individual IgE to IgG1 ratios. However, the mutated IgE_E_ and IgE_G_ clonotypes differed in the physiochemical properties of their unmutated counterparts (Fig. 4). In the BM, mutated and unmutated IgE_G_ significantly differed in their average net charges. In the mLN, mutated and unmutated IgE_G_ exhibited different aliphatic indexes and showed considerably different hydophobicities, and mutated and unmutated IgE_E_ exhibited different isoelectric points. The observed greater importance of antigen-binding properties in the BM compared to mLN are consistent with the idea that the former contains highly selected clonotypes.

These findings indicate that the differential binding of individual BCRs to antigens having distinct physiochemical properties has an impact on hypermutation and clonal selection, but does not determine the development of IgE_E_ versus IgE_G_.

### BCR signaling strength and IL-21 affect the ratio of IgE to IgG1 production

Next, we tested the possibility that differential BCR-signaling strength, which may mimic higher or lower affinities/avidities of BCRs for their cognate antigen, has an impact on the development of IgE versus IgG1. Therefore, we used a modified version of an advanced culture system that allows in vitro generation of germinal-center-like cells^46^. Naive B cells or sorted IgG1+ B cells were isolated from spleens of naive mice and stimulated with IL-4 and varying amounts of anti-IgM or anti-Ig kappa F(ab′)2 fragments. Anti-Ig kappa Ab stimulates both the IgM and IgD BCRs, which seem to be relevant for BCR activation by certain types of antigen^7^; and can also stimulate IgG1+ B cells.

**Fig. 5:**
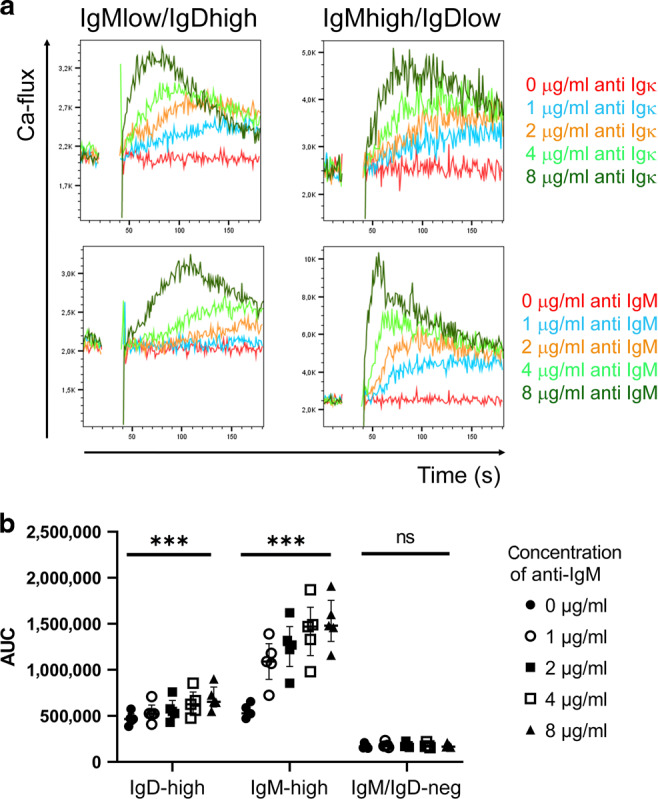
BCR crosslinking induced calcium flux. Single-cell suspensions from spleen were stained for IgM and IgD. After incubation with CalbryteTM 520 AM, separate samples were treated with various concentrations of polyclonal anti-IgM, or anti-Ig-kappa F(ab′)2, as indicated. Calcium flux was measured by flow cytometry (LSRII, BD, using a Flow Jo 10.7.1 software). **a** Representative histogram plots for IgMhigh/IgDlow naive B cells and IgMhigh/IgDlow marginal zone B cells, as indicated. **b** Statistical analysis of calcium flux after incubation with increasing concentrations of polyclonal anti-IgM F(ab′)2, for IgMhigh/IgDlow naive B cells, IgMhigh/IgDlow marginal zone B cells and IgMneg/IgDneg cells. Each symbol represents one cell culture well (*n* = 5 for all conditions). Representative data from one of three independent experiments, median and range are indicated. No sample was excluded from the analysis. Statistics: Friedmann-Test was calculated using R. ***p ≤ 0.001.

**Fig. 6:**
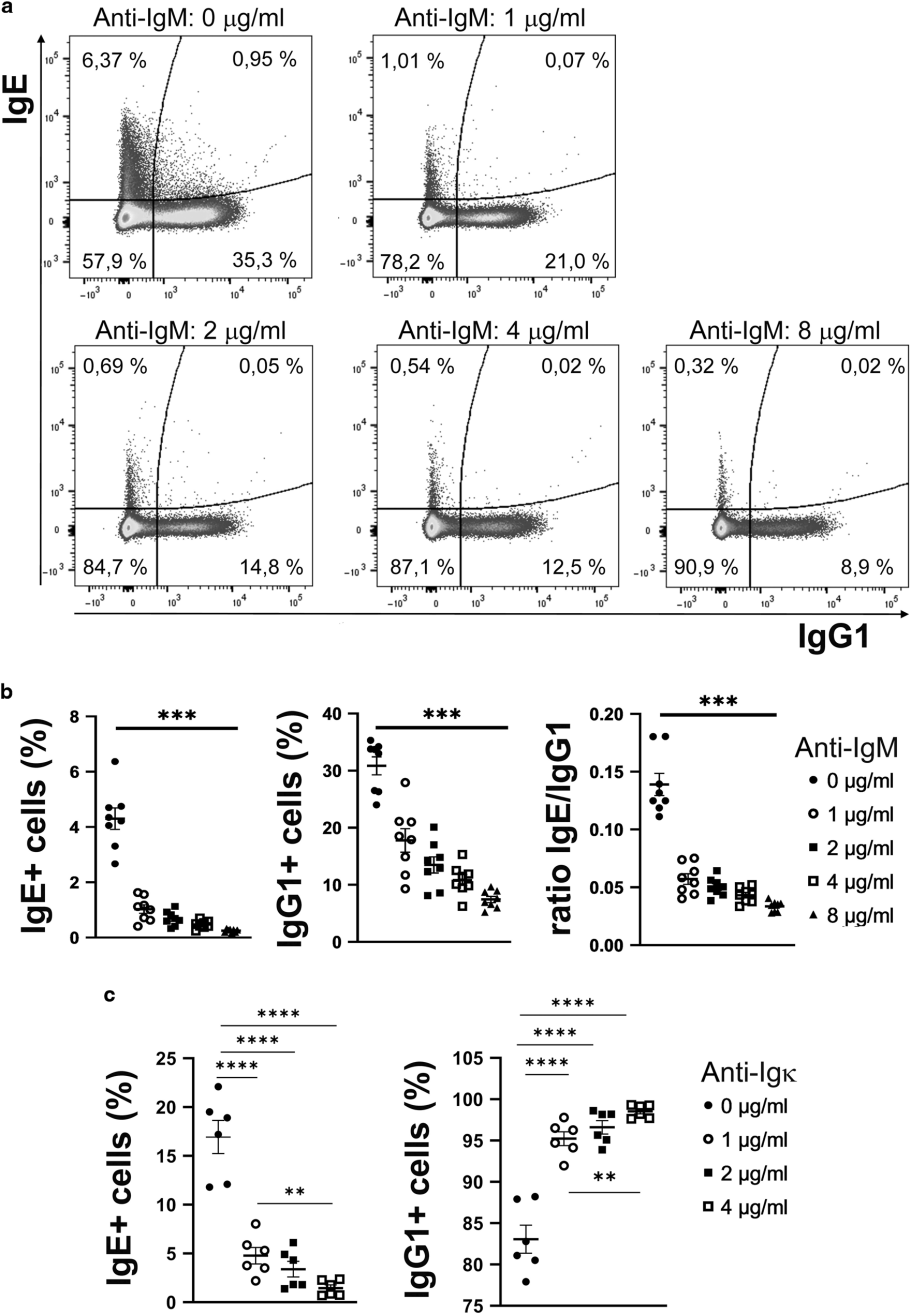
BCR stimulation alters the IgE to IgG1 ratio for class-switched cells. Naive B cells were cultured together with CD40L / BAFF-transfected feeder cells and IL-4, and stimulated with various concentrations of anti-IgM F(ab’)2 fragments. Subsequently, the frequencies of IgE+ and IgG1+ cells were analyzed by flow cytometry. Dead cells, debris, doublets, and feeder cells were excluded by Life/Dead stain and forward/sideward scatter and B cells were identified by CD19 expression. **a** Representative FACS plots of CD19+ B cells on day four of culture, stimulated with increasing concentrations of anti-IgM, as indicated. **b** Percentages of IgE+ and IgG1+ B cells, and the ratios between IgE:IgG1+ cells are shown at day four of culture, as indicated. **c** IgG1+ cells were sorted from B-cell cultures by FACS-sorting, stimulated with IL-4 and various concentrations of anti-Ig kappa F(ab’)2 fragments, re-cultured for another three days and subsequently analyzed by flow cytometry. Percentages of IgE+ and IgG1+ cells in cultures stimulated with various concentrations of anti-Ig kappa F(ab′)2 fragments, as indicated. Statistics: Each dot represents data from a culture of cells from one mouse (*n* = 8 for all conditions using anti-IgM; (*n* = 6 for all conditions using anti-Ig kappa). No sample was excluded from the analysis. **b** Data are pooled from three independent experiments. Data presented as mean ± SEM. Statistics: Friedman-test, ***p ≤ 0.001. C: paired *t*-test, **p ≤ 0.01; ****p ≤ 0.0001.

Both anti-IgM and anti-Ig kappa F(ab’)2 induced a dose-dependent calcium flux (Fig. 5), indicating that titrated concentrations of these reagents can mimic the differential signal strength that would characterize Ag stimulation of low or high-affinity BCRs. At day 4 of culture the frequencies of IgE+ and IgG1+ cells were analyzed by flow cytometry (Fig. 6a). With increasing BCR stimulation, the frequencies of IgE+ and IgG1+ cells decreased (Fig. 6b). The finding that IgG1+ and IgE+ cells were reduced is in accordance with the finding that BCR signaling can inhibit class switch to these Ig classes^47^. However, the decrease was greater for IgE+ cells and the ratio between IgE+ and IgG1+ cells dropped from approximately 0.14 in the absence of BCR stimulation, to below 0.03 at the highest concentration of stimulating antibodies. Therefore, we speculated that BCR stimulation could inhibit not only direct class switching from IgM to either IgG1 or IgE, but also the ability of IgM+ cells to switch sequentially to IgE via an IgG1+ intermediate, which could explain why IgE+ cells decreased more rapidly than IgG1+ cells. In line with this idea, sequential class switch-specific switch circles were reduced upon BCR stimulation (Fig. S4). Of note, switch circles for direct class switch were hardly detectable. This finding may indicate that in our cultures, sequential class switch might be the dominant mechanism of IgE formation. This is in line with earlier findings in B-cell cultures stimulated with LPS + IL-4^48^.

In order to further address the idea that BCR signaling inhibits sequential class switching, IgG1+ cells were sorted from cultures and re-cultured in the presence of IL-4 and increasing amounts of anti-kappa Ig F(ab’)2. After one day, without BCR stimulation, between 12 and 22 % of the isolated IgG1+ cells had switched to IgE and lost IgG1 expression. Increasing BCR stimulation inhibited the appearance of IgE+ cells in a dose-dependent manner but increased the percent of IgG1+ cells back to nearly 100 % (Fig. 6c).

These results extend the finding that BCR signaling inhibits class switch to IgG1 and IgE^47^ by showing that increasing BCR signaling also effects sequential class switching of IgG1+ cells to IgE, eventually reducing the ratio of IgE/IgG1+ cells.

**Fig. 7:**
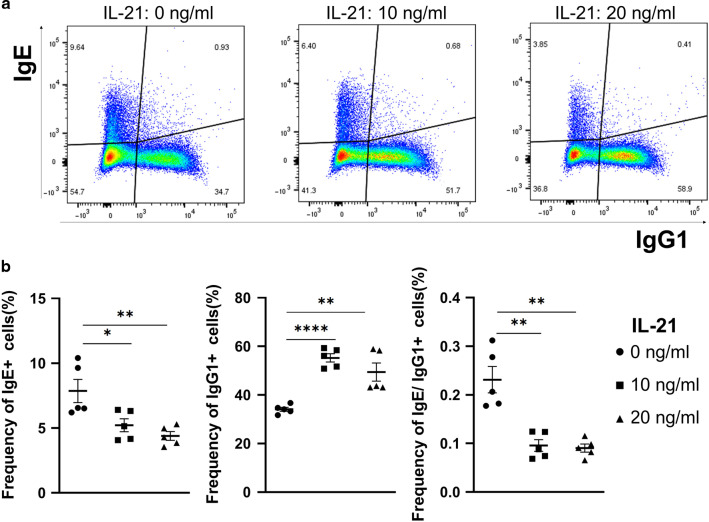
IL-21 reduces the IgE to IgG1 ratio for class-switched cells. Naive B cells were cultured together with CD40L / BAFF-transfected feeder cells and IL-4. At day 0 and day 1, various concentrations of IL-21 were added. The frequencies of IgE+ and IgG1+ cells were analyzed by flow cytometry at day 4. Dead cells, debris, doublets and feeder cells were excluded by Life/Dead stain and forward/sideward scatter and B cells were identified by CD19 expression. **a** Representative FACS plots of CD19+ B cells, stimulated with IL-21 in concentrations of 0, 10 or 20 ng/ml, as indicated. **b** Statistical analysis of the percentages of IgE+ and IgG1+ B cells, and the ratios between IgE:IgG1+ cells. Representative data from one of two independent experiments are shown. No sample was excluded from the analysis. Data presented as mean ± SEM. Statistics: Each symbol represents data from a culture of cells from one mouse (*n* = 5 for all conditions). Unpaired parametric *t*-test *p ≤ 0.05; **p ≤ 0.01; ****p ≤ 0.0001.

Antigen-binding affinities of individual BCRs also affect antigen-uptake^49,50^, which could modulate the interaction of antigen-specific B cells with T follicular helper cells which provide IL-21. Therefore, we tested the effects of IL-21 in our culture system. IL-21 promoted the formation of IgG1+ cells, while inhibiting IgE, eventually shifting the ratios of IgE+ to IgG1+ cells from approximately 0.1 to above 0.2 (Fig. 7a, b).

Together, these data suggest that BCRs could affect the ratios of individual B-cell clonotypes through both the strength of the BCR signaling, and via modulation of B-cell interaction with T follicular helper cells providing IL-21, at least in our murine models.

### IgG1+ cells with both low and high hypermutation rates can give rise to IgE

**Fig. 8:**
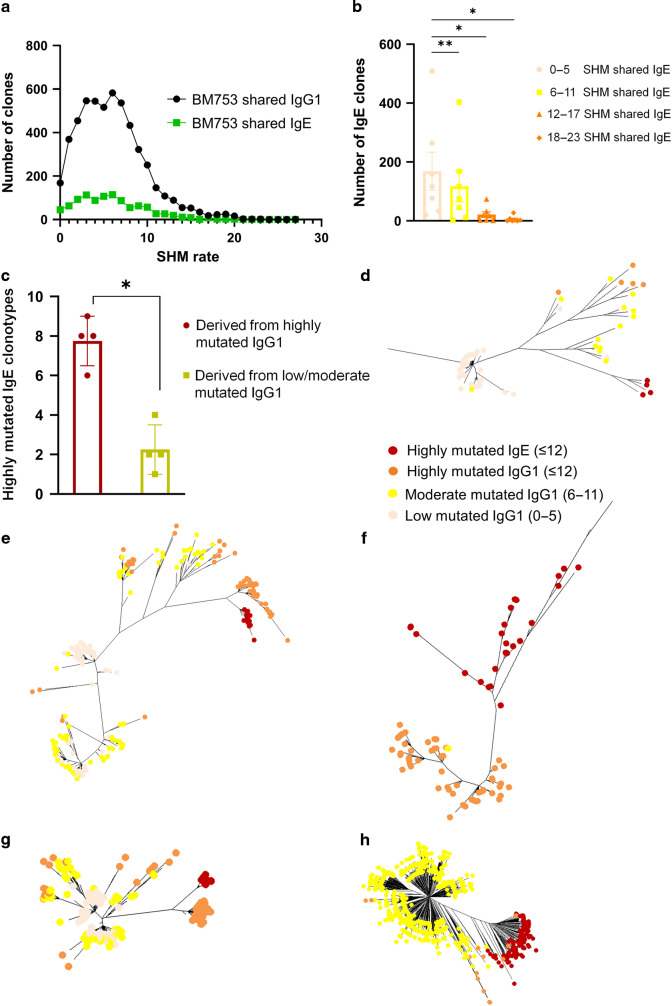
Clonal distribution of somatic hypermutation rates and lineage tree analysis. cDNA from allergic mice challenged o.g. 13 times with EG/EYP was analyzed by NGS. Individual clonotypes within the IgG1 and IgE IgH-repertoires were defined by unique VDJ-rearrangements. **a** Distribution of somatic hypermutation (SHM) rates of shared IgE and IgG1 clonotypes in one representative samples. **b** Statistical analysis of the distribution of somatic hypermutation of shared clonotypes in samples from BM of a total of 7 mice from two independent experiments (*n* = 7). Number of shared IgE clones with various hypermutation rates are shown, as indicated. **c** Analysis of lineages trees of highly mutated BM IgE clonotypes. Three samples containing less than 10 highly mutated IgE clonotypes were excluded from the analysis (*n* = 4). The number of highly mutated IgE (≥ 12 mutations) clonally related to highly mutated IgG1 (≥ 12 mutations) and low/moderately mutated IgG1 (≤ 11 mutations) are shown. (**b**, **c**) Each symbol represents data from one mouse and colored bars represent the mean. In total, lineage trees of 40 highly mutated IgE clonotypes were analyzed. **d**–**h** Examples of lineage trees of highly mutated IgE clonotypes are shown. Data presented as mean ± SEM. Statistics: Paired parametric *t*-test *p ≤ 0.05; **p ≤ 0.01.

In order to determine whether IgG1+ cells that had undergone extensive GC reactions could still undergo class switch to IgE, we analyzed the clonal distribution of hypermutation rates and mutational trees in our food allergy model. Most shared IgE clones showed low to moderate hypermutation rates below 12 mutations, less than 10% showed up to 27 mutations (Fig. 8a, b). Because highly hypermutated IgG1 clones cannot give rise to moderately mutated IgE clones, these results indicate that low to moderately hypermutated IgG1 clones contribute most of the sequential class-switched IgE.

However, lineage tree analysis indicated that approximately 80% of highly mutated IgE clonotypes (12 or more mutations) were related to highly mutated IgG1 clonotypes (Fig. 8c–h). Hence, class switch to IgE preferentially occurs before B cells have passed through extensive germinal center reactions, but highly mutated IgG1 clonotypes are still able to switch to IgE. The low proportion of highly mutated IgE derived from IgG1 progenitors with fewer mutations also indicates that once switched to IgE, the cells tend to avoid participating in further germinal center reactions.

Together, our findings indicate that the physiochemical properties of individual BCRs affect their mutation rates and clonal selection and that the BCR affects the ratio of IgG1 to IgE formation through its individual antigen-signaling strength and soliciting T-cell help. This mechanism could bias high-affinity B cells, receiving strong antigen-induced BCR signaling and IL-21 from T follicular helper cells, to favor IgG1 over IgE production.

## Discussion

Despite the great relevance of the production of mutated and unmutated IgE and IgG in allergy development, the mechanisms controlling the formation of these Ig subclasses are incompletely understood. Here we provide evidence that the physiochemical properties and signaling strength of BCRs control IgE and IgG1 formation and hypermutation on the level of individual B-cell clones.

In addition to the stronger direct inhibitory effects of antigen on B cells that express a high-affinity BCR, B cells with a high-affinity BCR can take up antigen very efficiently to present them to T cells, even at low antigen concentrations^49,50^. B cells with a high-affinity BCR may therefore receive stronger and/or prolonged help from T follicular helper cells, providing IL-21. Our results show that IL-21 can reduces the ratios of IgE to IgG1+ cells, at least in an in vitro model for the GC reaction. Based on these findings, we suggest a model in which strong antigen-signaling and optimized help from IL-21 producing T follicular helper cells bias high-affinity B cells to favor IgG1 over IgE production. Although our lineage tree analysis indicates that highly mutated IgG1 clones can still undergo class switch to IgE in vivo, the corresponding highly mutated IgE clones were very rare.

The antigen-binding priorities and polyreactivity of individual BCRs are determined by their physiochemical properties of the variable region^45^. The data presented in this paper imply that IgE-BCRs with distinct physiochemical properties are differentially subjected to hypermutation and clonal selection. Particularly mutated IgE_G_ showed a higher aliphatic index and hydrophobicity than unmutated IgE_G_ in egg-immunized mice_._ In this context it is interesting that many allergens are partly hydrophobic^51,52^, and the interaction of some proteins with lipids is suspected to increase their allergenic properties^53^. Taking into account that IgE_G_ clonotypes were much more abundant than IgE_E_ clonotypes and that hypermutated IgE but not unmutated IgE are believed to contribute to allergic symptoms^33^, the formation of hypermutated IgE_G_ might be most relevant for allergy development. If the hydrophobicity of allergens correlates with their capacity to promote the formation of hypermutated and highly pathogenic IgE remains to be elucidated.

Mutated IgE_E_ and IgE_G_ clonotypes also, on average, had lower PIs than their unmutated counterparts, indicating that the electric charge of an antigen/allergen could also have an impact on the relative production of these more or less allergenic IgE subtypes.

Since allergen-specific IgG can inhibit IgE-mediated mast cell activation, the ratio between IgE and IgG is expected to be another factor relevant for allergy development. The finding that individual BCR clonotypes vary considerably in proportion of IgE and IgG1 indicates that the ratio between the two Ig classes is controlled on the level of single B-cell clones and is influenced by the properties of individual BCRs. In contrast to what we observed for the IgE hypermutation rates, we did not find evidence that the IgE to IgG ratios are affected by the physiochemical properties of the BCR. Instead, antigen-binding affinities and avidities seem to affect the formation of IgE versus IgG1. Under physiological conditions, these two factors determine the BCR signaling strength. In our experiments we used titrated amounts of anti-BCR antibodies to induce differential BCR signals. Our results are in accordance with the finding of Jabara and colleagues showing that increased BCR signaling inhibits class switch from IgM to IgG1 and IgE^47^. The Jabara paper also showed that the effects of BCR stimulation on IgE and IgG1 were not due to altered proliferation or apoptosis. Extending these results, we found that increased BCR signaling can also inhibit class switch from IgG1 to IgE, eventually increasing the IgG to IgE ratios. Although the BCR stimulation-induced changes were not dramatic, they were in the same range as the differences between IgG1 and IgE serum titers in mice showing diarrhea and anaphylaxis compared to mice having no symptoms. This suggests that the increase in IgG to IgE ratio induced by increased BCR signaling could be relevant for the development of allergic symptoms, particularly when acting in synergy with IL-21. Under pathophysiological conditions, such increased BCR signaling could be permitted by high-affinity BCRs or BCRs that recognize highly polyvalent antigens.

That the ratios between IgE and IgGs are controlled on the level of single B-cell clones is a logical consequence, since IgGs can inhibit IgE-mediated anaphylaxis through neutralization of absorbed allergen^38^, a mechanism which may be most efficient for IgGs that have the same fine specificity as their IgE counterparts. Therefore, it is possible that high-affinity BCR clones may have a lower IgE to IgG ratio which may compensate for their increased allergic potential, particularly when allergen concentration is low, but this hypothesis needs to be investigated further.

While IgE production is normally short lived, even under conditions when IgG antibodies are maintained at high levels, it can persist in patients suffering from allergic diseases even without allergen re-exposure^10^. In our model, long-lasting IgE production was induced by repeated allergen challenges, which might reflect a common condition for allergic patients. Despite the extensive rounds of allergen challenges, the IgE compartment still contained a considerable proportion of unmutated IgE clones which co-existed with hypermutated and highly selected IgE clonotypes. This indicates that an extrafollicular response persisted despite an effective germinal center reaction. We found this somehow surprising, because mutated, affinity-selected germinal center-derived clones might be expected to outcompete the unmutated clones. Nevertheless, the selection processes in B-cell responses involving multiple and complex antigens are still incompletely understood^54^. In this context it is interesting that there is emerging evidence that germinal center responses and an extrafollicular response also co-exist in another chronic immune response, the autoantibody response in systemic lupus erythematosus^55,56^. In that disease, the two pathways of Ab production are driven by distinct T helper subsets^55,56^. It remains to be elucidated whether the same mechanism is also relevant in IgE-mediated allergic diseases.

In conclusion, our results imply that individual BCRs affect the overall nature and allergic potential of the IgE response. This finding may help to improve the efficiency of allergen-specific therapies through the use of allergens with altered physiochemical properties and/or binding valence.

## Methods

### Study approval

The animal experiments conducted in this study were done in strict accordance with the German regulations of the Society for Laboratory Animal Science (GVSOLAS) and the European Health Law of the Federation of Laboratory Animal Science Associations (FELASA). Protocols of the animal experiments performed at the University of Lübeck were approved by the respective local Committee on the Ethics of Animal Experiments of the state Schleswig-Holstein (Ministerium für Landwirtschaft, Umwelt und ländliche Räume des Landes Schleswig-Holstein). Approval numbers: 22-39_2016-06-17_Manz, and 122-39(46-6/18)_Manz. Animal experiments performed at CCHMC were approved by the CCHMC IACUC (approval number IACUC2020-0066).

### Mice

Female Balb/c mice were purchased from Charles River Laboratories (Sulzfeld, Germany) and maintained under pathogen-free housing conditions. Mice were housed according to standard operating procedures assuring mouse husbandry at the animal facilities of Cincinnati Children’s Hospital Medical Center and the University of Lübeck, with approval from the respective authorities. Animal caretakers regularly observed the mice for signs of disease, illness, or injury. If indicated, mice were observed by a veterinarian. After acclimatization period of at least one week, animal studies were performed with mice aged between 8–16 weeks at the start of the experiment.

### Experimental food allergy

Egg allergens were prepared and food allergy to hen’s egg was induced as described previously^39^. Briefly: EW and EY were separated and prepared as described. Mice were anesthetized by i.p. injection of 200 μL anesthetics (5 mg/mL Ketanest S, 1.5 mg/mL Rompun in DPBS), restrained and sensitized by intra-tracheal application of 40 µL EYP containing 50 µg EW. The procedure was repeated according to the sensitization schedules. For antigen (Ag) challenges, lyophilized EW was dissolved in sterile DPBS to a concentration of 500 mg/mL, then mixed with an equal volume of EYP. To assess diarrhea development, EW plus EYP was supplemented with food dye. Mice were intra-gastrically challenged by oral gavage with 300 µL of this mixture. Body temperature was measured by rectal thermometry (Physitemp). Potential confounders were minimized by co-housing the mice of all groups in shared cages and received identical treatments (groups were allocated at the end of the experiment according to the development of symptoms).

In order to reduce pain, suffering, and distress for the experimental mice, and to minimize the effects of potential confounders on the experimental results such as the spontaneous development of unwanted/unexpected diseases, criteria for exclusion of experimental mice were defined a priori. Experimental mice were observed once a week for general health conditions, conspicuous behavior, injuries, scrubby coat, conspicuous excrement or urine, signs of pain, belly stiffness, and paralysis. Exclusion criteria and humane endpoints were set a priory according to a scoring system, including signs of disease, illness, and weight loss, as described in detail in the animal applications, approved by the authorities. (see above). Criteria for exclusion were the development of unexpected symptoms, indicating the development of unexpected diseases such as infections or tumors. No mice had to be excluded because of these criteria. The sample size was decided on the basis previous experiments. Blinding was performed by assigning individual mice a mouse tag number. After allocation of groups according to allergic symptoms, these mouse tags were anonymously used during subsequent analysis.

### ELISA

To determine anti-EW and IgG1 levels, white Costar® 96-well plates were coated with 10 µg/mL EW in Tris/saline buffer (pH 7.2). For anti-EW-IgE levels plates were coated with 5 μg/mL anti-IgE mAb. Briefly, wells were washed and subsequently blocked with washing buffer containing 10% SuperBlock™(Thermo Scientific). Pooled serum samples of EW allergic mice were used as standard. Serum from naive mice were used as negative control. Standards (pooled serum samples from EW allergic mice) were serially diluted 1:2. After incubation with standards and diluted samples, wells were repeatedly washed and subsequently incubated with biotinylated anti-mouse IgG1 (Southern Biotech) or for the detection of EW-IgE incubated with biotinylated EW. Wells were then washed and incubated with Streptavidin-HRP (Thermo Scientific). Following further washing, substrate (Thermo Scientific) was added to the wells and responses were immediately measured with a Luminometer (FlUOstar Omega, BMG Labtech). Students performing the ELISA assays were not aware about the group allocation.

### NGS analysis of the IgE and IgG1 repertoire

Tissue samples were collected from BM and mLN samples, cut into small pieces (2 × 2 mm), transferred into tubes with RNAlater and incubated at 4 °C overnight, then stored at −20 °C in RNAlater solution (Qiagen). Later, RNA was isolated using the RNeasy mini Kit (Qiagen #74104). Reverse transcription of the RNA samples was performed using the Qiagen-One-Step-RT-PCR Kit (Qiagen). The custom-designed primers specific for murine IgG1 and IgE were synthesized by iRepertoire. PCR product was further amplified using generic primers provided in the iRepertoire kit that bind Illumina-adapters introduced by the primers used in PCR 1. For the subsequent amplification the Qiagen Multiplex PCR kit (Qiagen #206143) was used. A quality control was performed using an Agilent Bioanalyzer 2100 and an RNA 6000 Pico kit (Agilent). Based on the integrity of ribosomal RNA, RNA-integrity numbers (RIN) were determined (RIN = 10 meaning intact RNA, RIN = 0 completely degraded RNA). Only RNA samples with RIN of 6 or higher were used for NGS analyses.

For preparation of the library for paired-end sequencing with the Illumina MiSeq system 5 μL aliquots of each sample at a concentration of 2 nM (diluted with Tris/HCl based on concentration determination as described above) were pooled and PhiX Control v3 was added to the library as an internal sequencing control. Approximately 1–1.5 million sequencing reads per sample of the BCR library were obtained.

The quality of the NGS data was determined using FastQC. The criteria for sample exclusion were set a priory: samples showing a median quality score for any base of less than 20 units were excluded from further analysis. Due to insufficient cDNA quality, one BM sample of an allergic mouse and one BM sample of a naive mouse were excluded from NGS analysis.

The data was de-multiplexed into the individual samples with ClonoCalc (153). The determination of clonotypes (unique CDR3 nucleotide sequences) was performed using MiXCR (168). The output data included a clone ID that was given by the program sorted by occurrence of this sequence starting at 1 for the most abundant clonotype, abundance of the clonotype (number of sequences, percentage of this clonotype of all sequences), aligned V-, D-, J- and C-gene (the C-gene alignment allows separation of sequences into IgE and IgG1), CDR3 nucleotide and amino acid sequence, the quality score, and length of the CDR3-region. Additionally, to avoid artificial diversity due to PCR errors, all clonotype sequences that appeared only once were removed. Overlapping sequences between IgE and IgG1 were determined using InteractiVenn.

For analysis of clone sizes, identification of shared clonotpes and later specification of these clones, each mouse cDNA FASTA sequence from MIXCR or IRepertoire (iRepertoire, Inc. AL, USA) was separated into IgE and IgG1 FASTA sequences. IgE and IgG1 FASTA files were uploaded to the international ImMunoGeneTics (IMGT) database^57^. The resultant IMGT/HighV-QUEST output file for IgE and IgG1^58,59^ were used as input files in the IMGT/StatClonotype database to generate their corresponding IMGT/StatClonotype text files^60,61^. IgE and IgG1 V-REGION-nt-mutation-statistics.txt file from IMGT/HighV-QUEST and IMGT/StatClonotype.txt file from each mouse were used for further downstream analysis in an Excel VBA Macro (https://mega.nz/file/wwBgAZBD#yrZcvHVpLINE6GYmrEeRDSWiU6XpIeCqnlj425uvzhA). The VBA macro excludes non-productive clones, clones with one copy, clones without a conserved CDR3-IMGT anchors (cysteine C 104, tryptophan W 118 or phenylalanine F 118), and clones with no V, D, or J call. The macro defines a clone as a unique VDJ gene rearrangement with conserved anchors. From here, the macro further evaluates the shared and unique VDJ rearrangement between IgE and IgG1, the bias to IgE and IgG1 within the shared clones, the mutated and unmutated clones based on the presence or absence of mutation in the V gene.

For the analysis of the lineage trees, the V region nucleotide sequences from highly mutated IgE clones and its shared IgG1 counterparts were aligned by ClustalW^62^, and the lineage trees were generated using DNA maximum parsimony criterion. The resultant tree was viewed as a radiation tree with Mega 11^63^. In each mouse, the mutation table of the shared IgE and IgG1 clones was used to plot the mutation distribution in graph pad prism. Potential confounders are minimized by comparing the properties IgG vs IgE subtypes (IgEE, IgEG, etc.) in-between LN and BM samples from the same individual mouse, and in-between IgG vs IgE subtypes within the same sample, excluding cage effects and effects of differential treatment.

### B-cell culture

B cells were isolated from single-cell suspensions of the spleen using the EasySep™ Mouse Pan-B-Cell Isolation Kit (Stem Cell) and co-cultured together with mitomycin C M0503 (Sigma-Aldrich) treated 40LB feeder cells, a CD40-ligand (CD40L)- and B-cell activating factor (BAFF)-transfected fibroblast cell line as described^64^. At the start of the culture, B cells were stimulated with IL-4 together with various concentrations of polyclonal goat anti-mouse IgM F(ab′)2 fragments (Dianova), or anti-Ig kappa F(ab′)2 fragments (Sigma-Aldrich). To investigate the role of IL-21, recombinant murine IL-21 (ImmunoTools) was added at various concentrations at days 0 and 1. On day 4, B cells were isolated from the culture, stained for IgG1 (clone: RMG1-1), IgE (clone: RME-1), IgM (clone: RMM-1), IgD (clone: 11-26c.2a), CD138 (clone: 281-2), and CD19 (clone: 6D5, all Biolegend), and analyzed by flow cytometry. Dead cells and feeder cells were excluded from the analysis using the Fixable Viability Dye eFluor 780 (eBioscience), and FSC-A versus FSC-H and SSC-W versus SSC-H gating. For analysis of sequential class switch from IgG1 to IgE, IgG1+ B cells were FACS sorted from 4 day cultures or directly from fresh spleens (CAnaCore, cell sorting core facility of the University of Lübeck). Flow cytometry analysis was performed using an LSRII (BD). Data were analyzed using the Flow Jo 10.7.1 software. As a quality control for efficient B-cell stimulation, cell expansion was assessed under the microscope and/or by cell counting. Cultures with inefficient cell expansion, i.e. ≥ 3-fold expansion of B-cell numbers till day 4, were not used for analysis. In order to minimize potential confounders further, B cells isolated from individual mice were spitted and used to study the impact of various culture conditions. The outcome measures used to determine the sample size were the frequencies of IgE+ and IgG1+ cells. Sample sizes were planned on the basis of preliminary experiments. Since the flow cytometric analysis of the cell cultures is objective, analysis of the cell cultures was not blinded.

### Statistical analysis

Data analysis was performed using GraphPad PRISM software, statistical tests are indicated in individual figure legends. NGS datasets were tested for normality using the D’Agostino & Pearson omnibus normality test. If appropriate, nonparametric tests were used and indicated in the respective figures. *p < 0.05, **p < 0.01, and ***p < 0.001. Data were plotted as mean ± SEM. A *p* value < 0.05 was considered significant.

## Supplementary information

Supplementary Figures and tables

## Data Availability

Datasets related to this article can be found at: https://www.scidb.cn/s/UFfyua.
