# Supplementary material for: B-cell receptor physical properties affect relative IgG1 and IgE responses in mouse egg allergy
Source: Mucosal Immunol. 2022 Sep 16;15(6):1375–88. doi: 10.1038/s41385-022-00567-y (PMC9705252; doi:10.1038/s41385-022-00567-y)
Supplement: Supplementary file 1 — Supplementary Figures and tables [file 41385_2022_567_MOESM1_ESM.pdf]

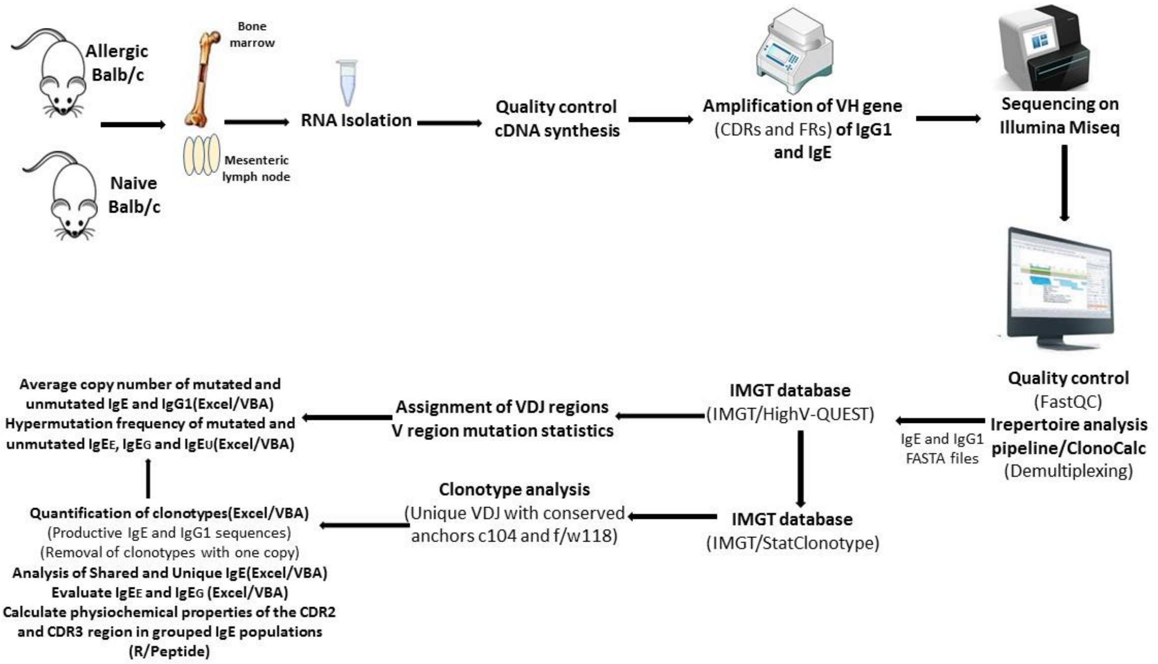

Figure S1. NGS work flow. BM and mLN were taken from fife mice with maintained symptoms of food allergy (temperature drop ≥ 1°C and diarrhoea in response to challenges; "allergic mice") and two similarly housed mice that have never been in contact with EW/EYP ("naïve" or "control"). RNA was isolated and cDNA synthesised according to the Method section, using primers specific for conserved regions in the V-gene on one side and for the constant region of IgE and IgG1 respectively allowing an amplification of the CDR3 region as well as parts of CDR1, FR2, CDR2 and FR3. The NGS data quality was determined using FastQC and de-multiplexed into the individual samples with ClonoCalc. Due to insufficient quality, one BM sample form an allergic mouse and one BM sample from one naïve mice were excluded from further analysis. Clonotypes were determined from the IMGT/STAT clonotype output data as productive sequences with a unique VDJ and conserved anchors (Cysteine 104 and Phenylalanine/Tryptophan 118).

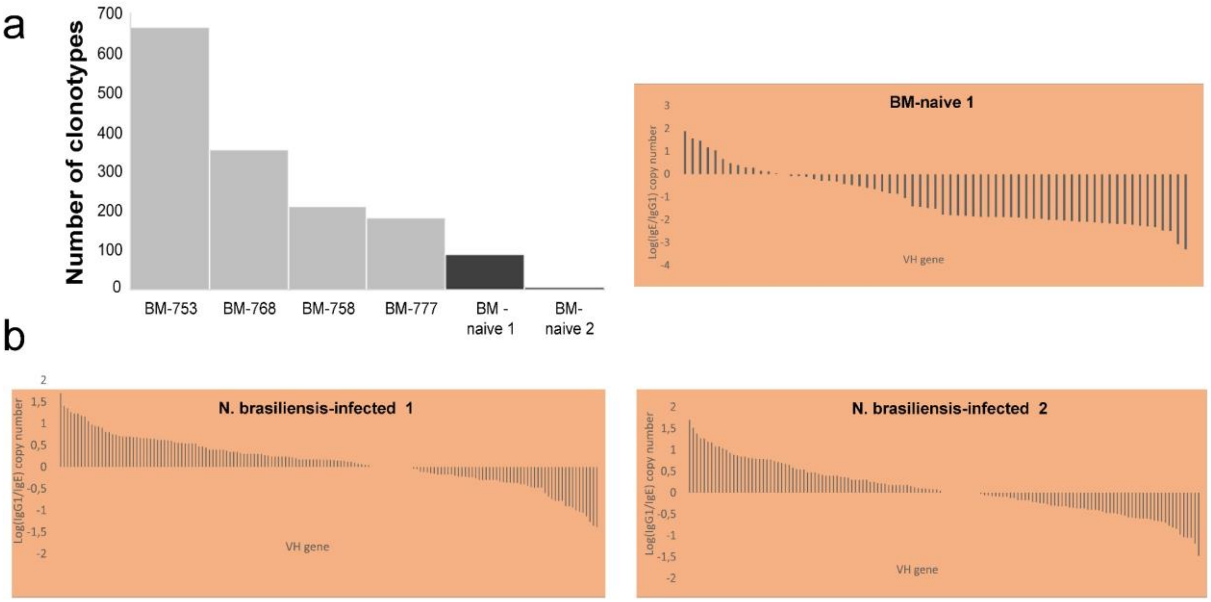

Figure S2. IgE and IgG1 compartments in naïve and helminth-infected mice. (a) VDJ IgH-regions in BM of two naïve mice were analysed by NGS. Left: number of individual clonotypes in the BM of naïve mice (BM-naïve 1; BM-naïve 2) in comparison to sequences from BM of four food allergic mice (BM-753; BM-768; BM-758; BM-777). Right: Ratio between IgE and IgG copy numbers of individual clonotypes of BM- from two naïve mice. Each bar represents an individual clonotype. (b) Published sequences from isolated BM plasma cells from mice infected twice with N. brasiliensis were reanalysed [1] Ratio between IgE and IgG copy numbers of individual clonotypes of two N. brasiliensis-infected mice. Each bar represents an individual clonotype.

Literature

1. Turqueti-Neves A, Otte M, Schwartz C, et al. The Extracellular Domains of IgG1 and T Cell-Derived IL-4/IL-13 Are Critical for the Polyclonal Memory IgE Response In Vivo. *PLoS Biol.* 2015;13(11):e1002290.

a

Sample ID	Shared IgE				Unique IgE			
	Mutated IgEG	*Unmutated IgEG*	*Mutated IgEE*	*Unmutated IgEE*	*Mutated IgEU*	*Unmutated IgEU*	*Total IgEU*	*Highly expanded IgEU*
BM82400	39	4	13	4	20	1	21	4
BM82416	10	1	1	0	5	0	5	3
BM777	120	8	9	1	22	1	23	0
BM758	149	12	11	5	20	1	21	0
BM82439	10	0	2	0	3	0	3	3
BM753	449	18	41	11	78	10	88	1
BM768	196	11	35	1	65	1	66	4
mLN768	818	68	321	61	465	22	487	1
mLN758	273	19	32	8	59	1	60	0
mLN769	510	36	52	10	97	2	99	1
mLN753	601	86	173	29	276	14	290	0
mLN777	621	58	89	20	121	13	234	5

b

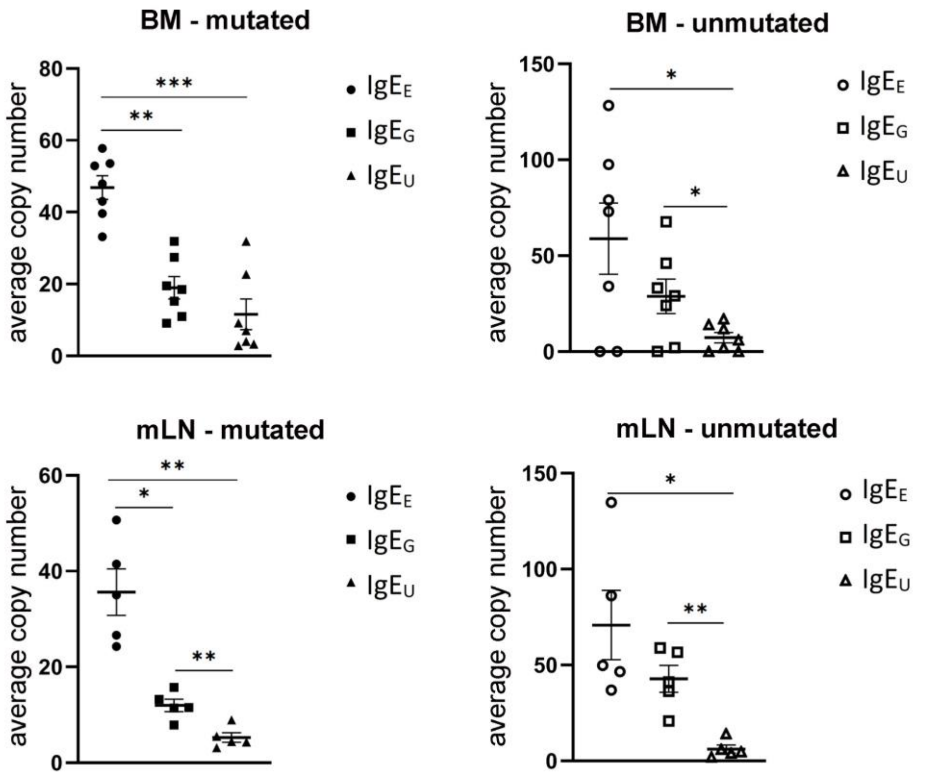

Figure S3. IgE_U copy numbers and highly expanded clones IgE_U. (a) Table showing the numbers of mutated, unmutated, shared and unique IgE clonotypes and the numbers of highly mutated clones among IgE_U, in individual samples from BM and mLN, as indicated. (b) Shared clones were classified as IgE-biased (IgE_E, with an IgE/IgG1 copy number ratio ≥ 2) or IgG1-biased (IgE_G, with an IgG1/IgE copy number ratio ≥ 2). Average copy numbers of IgE_E, IgE_G and unique IgE (IgE_U) in BM and mLN.

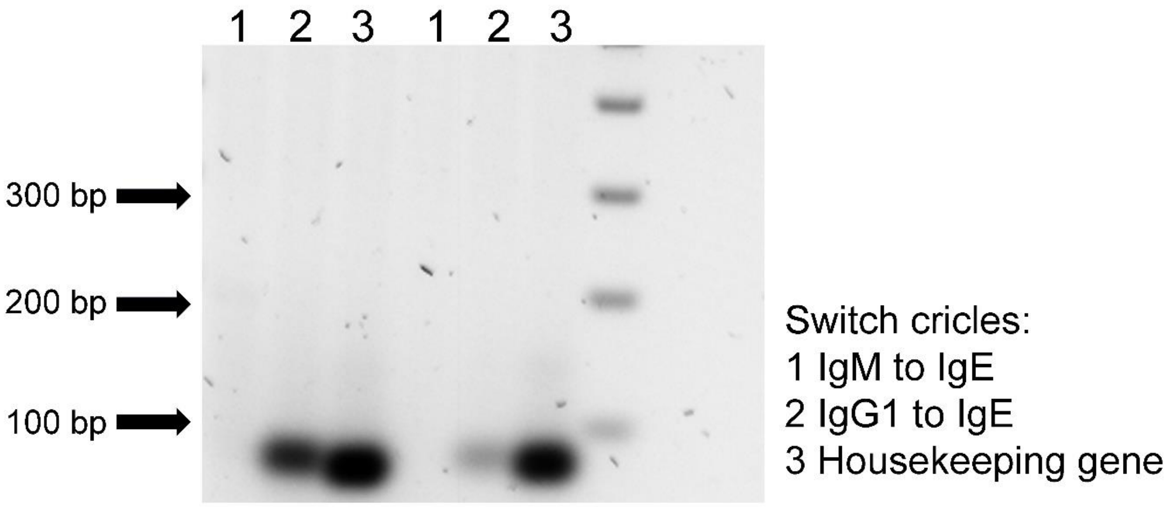

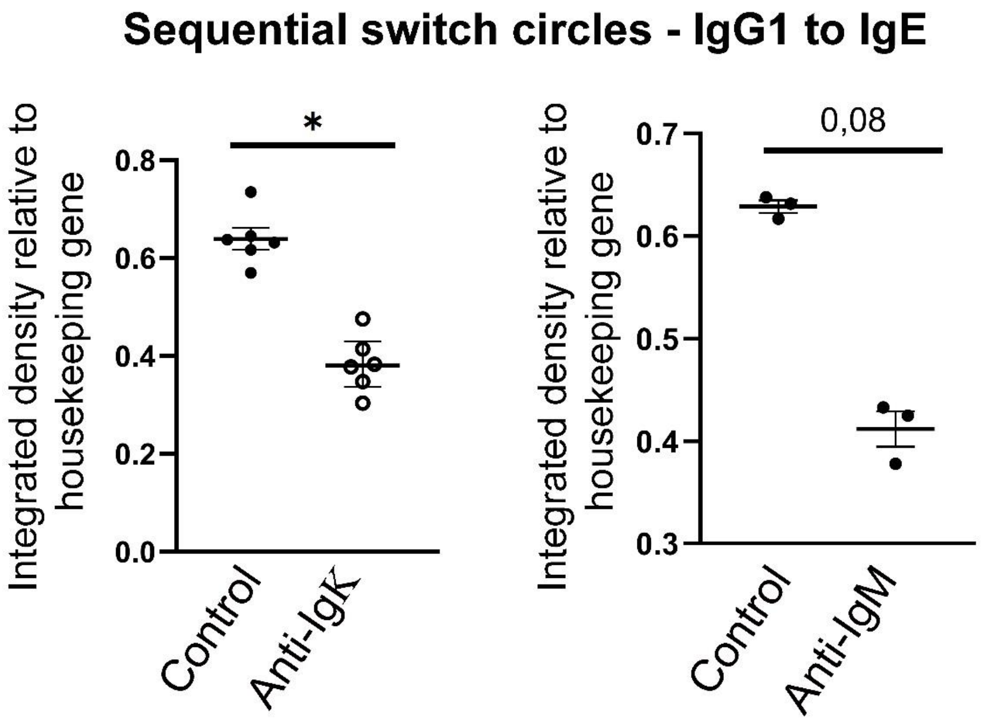

Figure S4. Sequential class switch specific circles. Splenic B cells were co-cultured for 3 days with CD40L and BAFF transfected fibroblasts and IL-4, and left untreated (Control) or treated with 8 ug/ml anti-Ig kappa F(ab')2 fragments or anti-IgM F(ab')2 fragments, as indicated. Upper panel shows representative PCR gel. Lower panel: quantification and statistical analysis. Each dot represents one sample. Mean and SEM are shown. Data for anti-Ig kappa F(ab')2 fragments are pooled from two independent experiments. Statistics: Wilcoxon signed rank test was calculated using R. *p ≤ 0.05.

Table S1. Number of IgE and IgG1 clones and sequences analysed in the egg allergy model

Mouse I.D	IgE clones	IgG1 clones	IgE sequences	IgG1 sequences
BM2	6	406	680	94063
BM4	90	660	19325	136162
BM753	666	2627	25179	394159
BM758	210	1982	8867	294907
BM768	355	1617	17098	201506
BM777	181	2105	6077	320180
mLN2	295	1562	39551	279335
mLN4	382	1858	19931	154151
mLN753	1475	2340	80637	225155
mLN758	474	1979	33622	150335
mLN768	2240	2834	170935	331475
mLN769	827	2376	39542	291774
mLN777	1050	2774	49160	313729

Table S2. IgE clonotypes in N brasiliensis infected mice

sample I.D	Infection	mutated IgE_G	unmutated IgE_G	mutated IgE_E	unmutated IgE_E	mutated IgE_U	unmutated IgE_U
BM1	Primary	2	1	8	2	32	9
BM2	Primary	3	0	7	0	43	12
BM1	Secondary	35	0	57	3	263	4
BM2	Secondary	38	3	47	5	253	1

Re-analysis of sequences from isolated BM plasma cells after primary or secondary infection with N. brasiliensis, originally described by Neves et al. [1]. Clonotypes present in the IgE repertoire but absent from the IgG repertoire are defined as "unique IgE clones". Shared clones were classified as IgE-biased (IgE/IgG copy number ratio of ≥ 2), or IgG1-biased (IgG/IgE copy number ratio of ≥ 2).

Literature

1. Turqueti-Neves A, Otte M, Schwartz C, et al. The Extracellular Domains of IgG1 and T Cell-Derived IL-4/IL-13 Are Critical for the Polyclonal Memory IgE Response In Vivo. *PLoS Biol.* 2015;13(11):e1002290.
